# Callus cell proliferation from broccoli leaf slice using IBA and BAP *in vitro* culture: Its biochemical and antioxidant properties

**DOI:** 10.1016/j.dib.2015.11.061

**Published:** 2015-12-12

**Authors:** A.B.M. Sharif Hossain, Imdadul Haq, Nasir A. Ibrahim, Mohammed Saad Aleissa

**Affiliations:** a Program of Biotechnology, Department of Biology, Faculty of Science, University of Hail, Saudi Arabia; b Program of Biotechnology, ISB, Faculty of Science, University of Malaya, Kuala Lumpur, Malaysia; c Department of Biology, Faculty of Science, AI-imam Muhammad Ibn Saud Islamic University, Saudi Arabia

## Abstract

Plant tissue or cell culture keeps a significant role in micro-propagation in the plant production industry. Combination of 6-Benzylaminopurine (BAP) and other plant growth regulators like 1-Naphthaleneacetic acid (NAA) or Indole-3-acetic acid (IAA) or indole-3-butyric acid (IBA) was used in the most of the research in tissue culture. The study was carried out to investigate the optimization of the concentration of IBA and BAP combination (0, 0.25, 0.50, 1.0, 1.50, 2.0, 2.5, 3.0 and 3.5 mg/l) for the root, callus and leaf proliferation from the leaf cutting slice. The highest number (6.75) of root proliferation was observed in the concentration of 2.0 mg/l IBA+0.25 mg/l BAP combination. The callus initiation was found in the concentration of IBA 1.0–3.5 mg/l+BAP 1.0–2.0 mg/l. However, the highest callus weight was observed at the concentration of IBA 1.5 mg/l+BAP 1.0 mg/l combination than other combination of concentrations. Positively leaf initiation and formation was better in the concentration of IBA 1–3.5 mg/l+BAP 1.0–2.0 mg/l combination. In addition, the 2,2-diphenyl-2-picrylhydarzyl (DPPH) free radical scavenging potential was higher (70.1%) in leaves extract than in callus extracts (46.3%) at the concentration of 10 mg/ml though both extracts had lower DPPH free radical scavenging activity compared to the positive control, vitamin C and BHT. Theresults conclude that the optimum concentration was IBA 1.5 mg/l+BAP 1.0 mg/l combination to produce callus cell proliferation and concentration of 2.0 mg/l IBA+0.25 mg/l BAP combination was the optimum for root proliferation of broccoli *in vitro*.

**Specialization Table**

**Table:** 

Subject area	Biology
More specific subject area	Plant cell and tissue culture Biotechnology
Type of data	Table and Figure
How data was acquired	Culture in Growth chamber, antioxidant activity, DPPH free radical measured by spectrophotometer.
Data format	Raw data collection and analyzed.
Experimental factors	Single factor
Experimental features	10 replicates were used as CRD design
Data source location	Hail city, Saudi Arabia and Kuala Lumpur, Malaysia
Data accessibility	Data are presented in this article.

**Value of the data**

1. The data provides the information of the effect of different concentrations of IBA (auxin) and BAP (cytokinin) on the leaf, root and callus proliferation from broccoli leaves slice *in vitro* culture.
2. This data would be valuable for further studies of physiological, biochemical and antioxidant activity in broccoli explants *in vitro* culture.

## 1 Data

**Fig. 1.1:**
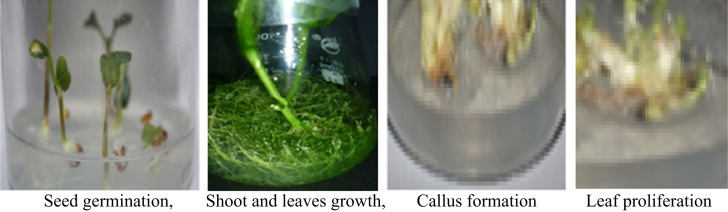
Photo shows the callus and explat from leaf cutting slice of broccoli.

**Fig. 1.2:**
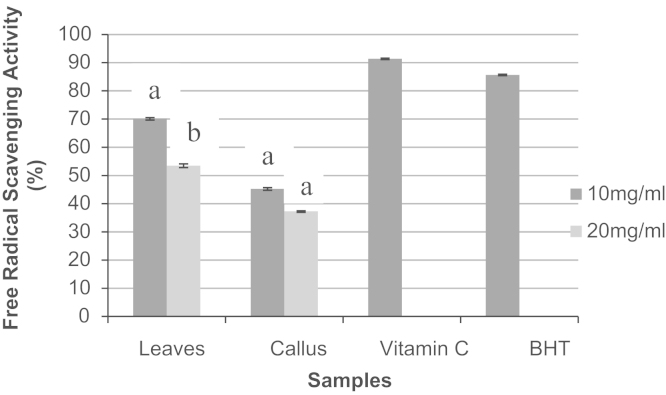
The antioxidant activity of the selected parts (leaves and callus) of broccoli at concentration of 10 and 20 mg/ml. Mean±SE (*n*=10). The same letters are not statistically different at 5% level of significance by Least Significant Difference (LSD) test.

In the data, the effects of IBA and BAP on the callus, roots and leaf formation have been shown (Table 1.2). In Table 1.3, effects of different combinations of hormones on the fresh weight of callus produced have been mentioned. Furthermore, measurement of the OD reading of control, positive control (vitamin C and BHT) and other samples have been taken at 515 nm using spectrometer (Table 1.4). Fig. 1.1 has shown the callus and explants from leaf cutting slice of broccoli. Moreover, Fig. 1.2 has represented the antioxidant activity of the selected parts (leaves and callus).

## 2 Materials and methods

### 2.1 Preparation of Murashige and Skoog (MS) basal media

The MS basal media [1] were used as control and seed germination was prepared following the standard procedures for MS powder form preparation (Table 1.1). MS powder form was added in a beaker with 800 ml distilled water followed by 30 g of sucrose and 2.8 phyta gels and adjusted the pH to 5.8 so that the final volume of the medium was 1000 ml.

### 2.2 Media in the autoclave

MS basal media with auxin was prepared by adjusting the pH to 5.8 by using 1 N HCl and 1 M NaOH. Then, the media was fractional in 30 ml and was added into jam jars (7×4.5 cm^2^) and autoclaved at 15 psi and 121 °C for 20 min. After that, the sterilized media were cooled and kept in culture room under dark condition. Preparation of media was completed a week before use to reduce water condensation in jam jars and the media was sterilized completely.

### 2.3 Seed sterilization and germination in the MS media

Seeds of *broccoli* were obtained from the nursery. A total of 100 seeds were used on MS [1] basal medium. The 20 jam jars were used to culture the seeds and five seeds were germinated on every jam jars. The seeds were washed in 70% ethanol for about 5 min, and then rinsed in 15% chlorox for 15 min. The seeds were brought into the laminar flow hood and further rinsed with sterile DH20 for a few seconds. Then, the sterilized seeds were germinated on MS basal media for 7 days. This process was carried out under aseptic condition in the laminar flow. The seeds were exposed to light from cool white fluorescent tubes for a photoperiod of 16 h in the incubation room at 25–28 °C.

### 2.4 MS basal media with IAA and IBA and BAP (2nd time media preparation)

The MS media with IBA and BAP were used as rooting media, MS powder form was added in a beaker filled with 800 ml distilled water and 30 g of sucrose was added. Then, the hormones with specific concentration were added. The pH was similarly adjusted and 2.8 g phyta gel was added, so that 1000 ml of medium was prepared. The media with hormones were prepared for 10 replicates of each hormone concentration. The BAP (as cytokinin) and IBA (as auxin) concentrations were 0, 0.25, 0.5, 1.0, 1.5, 2.0, 2.5, 3 and 3.5 mg/l.

### 2.5 Leaf cutting slice culture on MS supplemented with IBA and BAP

After one week of germination, 7 days seedlings were selected as a source of explants. The explants leaves were cut and transferred into the media with different concentrations of IBA and BAP 0, 0.25, 0.5, 1.0, 1.5, 2.0, 2.5, 3.0, 3.5 mg/l. Each treatment was consisted as five replications. Broccoli leaves were sliced in the clean bench. After that, *in vitro* culture on MS basal was performed. The leaf slice cultures were put in the growth chamber in the incubation room at 25–28 °C. Randomized complete block designed (RCBD) was used during sampling setting.

### 2.6 Antioxidant activity of broccoli

The antioxidant was evaluated based on the scavenging activity on the stable 2,2-diphenyl-2-picrylhydarzyl (DPPH) free radical measured by spectrophotometer [2]. DPPH was useful reagent for investigating the free radical activities of compounds. A freshly prepared DPPH solution was exhibited a deep purple color with maximum absorption at 515 nm. The DPPH observation was a non-enzymatic method currently used to provide basic information on the ability of extracts to scavenge free radical. The OD reading of control, positive control (vitamin C and BHT [3]) and all samples were taken at 515 nm using spectrometer. The use of the percentage of free radical scavenging activity was calculated by the following formula for vitamin C. Vitamin C=[(A control−A sample)/A control]x100 [3].

### 2.7 Data collection

Root and callus formation and leaf proliferation were observed and data were collected after one month of treatment setting. The 2,2-diphenyl-2-picrylhydarzyl (DPPH) free radical was measured.

### 2.8 Design and statistical analysis

Randomized block design was used during sampling setting. Standard deviation and then standard error was made to compare the replicates. Least Significant Difference (LSD) test was used for data analysis.

## Figures and Tables

**Table 1.1:** Standard procedures for MS media preparation.

Component	Unit
MS powder form with vitamin	4.4 g
Sucrose	30 g
Phyta gel	2.8 g
pH	5.8

**Table 1.2:** Effects of IBA and BAP on the roots, leaf and callus formation from broccoli leaf slice.

IBA	BAP	No. of root formation	Observation of callus	Leaf proliferation
0	0	0	−	−
0	0.25	0.25±0.25	−	−
0	0.5	0	−	−
0	1.0	0	−	−
0	1.5	0	−	−
0	2.0	0	−	−
0	2.5	0	−	−
0	3.0	0	−	−
0	3.0	0	−	−
0.25	0.25	1.0±0.41	−	−
0.25	0.5	1.75±0.25	−	−
0.25	1.0	2.0±0.41	−	−
0.25	1.5	3.5±0.65	−	−
0.25	2.0	2.5±0.29	−	−
0.25	2.5	2.0±0.41	−	−
0.25	3.0	2.25±0.25	−	−
0.25	3.5	2.25±0.25	−	−
0.5	0.25	1.5±0.29	−	−
0.5	0.5	1.25±0.25	−	−
0.5	1.0	1.5±0.65	−	−
0.5	1.5	1.75±0.48	−	−
0.5	2.0	2.0±0.71	−	−
0.5	2.5	1.75±0.48	−	−
0.5	3.0	1.5±0.29	−	−
0.5	3.5	1.25±0.25	−	−
1.0	0.25	1.75±0.49	−	−
1.0	0.5	1.25±0.49	−	−
1.0	1.0	1.5±0.29	Callus initiated	+
1.0	1.5	1.5±0.29	Green and whitish callus	+
1.0	2.0	1.75±0.48	Compact and globular callus	+
1.0	2.5	1.5±0.29	−	
1.0	3.0	2.0±0.41	−	−
1.0	3.5	3.25±0.75	−	−
1.5	0.25	2.75±0.48	−	−
1.5	0.5	3.5±0.65	Callus initiated	−
1.5	1.0	2.5±0.65	Green and whitish callus	+
1.5	1.5	2.75±0.48	Compact and globular callus	+
1.5	2.0	1.5±0.65	−	+
1.5	2.5	2.25±0.48	−	−
1.5	3.0	2.25±0.25	−	−
1.5	3.5	2.0±0.41	−	−
2.0	0.25	6.75±0.49	−	−
2.0	0.5	3.5±0.65	−	−
2.0	1.0	2.5±0.29	Callus initiated	+
2.0	1.5	3.5±0.65	Green and whitish callus	+
2.0	2.0	2.5±0.29	Compact and globular callus	+
2.0	2.5	3.0±0.41	−	−
2.0	3.0	2.5±0.29	−	−
2.0	3.5	2.75±0.25	−	−
2.5	0.25	2.5±0.65	−	−
2.5	0.5	2.25±0.25	−	−
2.5	1.0	2.5±0.29	Callus initiated	+
2.5	1.5	4.0±0.41	Green and whitish callus	+
2.5	2.0	2.0±0.41	Compact and globular callus	+
2.5	2.5	3.0±0.71	−	−
2.5	3.0	2.5±0.29	−	−
2.5	3.5	2.5±0.29	−	−
3.0	0.25	2.0±0.41	−	−
3.0	0.5	4.0±0.41	−	−
3.0	1.0	2.5±0.29	Callus initiated	+
3.0	1.5	2.75±0.25	G Green and whitish callus	+
3.0	2.0	3.5±0.65	Compact and globular callus	+
3.0	2.5	3.0±0.41	−	−
3.0	3.0	2.5±0.29	−	−
3.0	3.5	2.75±0.63		−
3.5	0.25	2.75±0.48	−	−
3.5	0.5	4.0±0.41	−	−
3.5	1.0	3.25±0.48	Callus initiated	+
3.5	1.5	2.5±0.29	Green and whitish callus	+
3.5	2.0	2.75±0.48	Compact and globular callus	+
3.5	2.5	2.5±0.29	−	−
3.5	3.0	3.0±0.41	−	−
3.5	3.5	2.5±0.29	−	−

Mean±SE (*n*=10). + = organ (leaf) formation was indicated. − = no-indication of organ formation.

**Table 1.3:** Effects of different combination of hormone on the fresh weight of callus produced from leaves.

BAP	IBA	Callus weight
1.0	0.25	0.6±0.15
0.5	1.02±0.06
1.0	1.4±0.09
1.5	3.9±0.15
2.0	2.35±0.23
2.5	1.8±0.11
3.0	2.15±0.07
1.5	3.5	2.18±0.4
0.25	2.7±0.15
0.5	2.4±0.04
1.0	2.37±0.12
1.5	2.45±0.10
2.0	2.3±0.09
2.5	2.35±0.06
3.0	2.0±0.17
2.0	3.5	2.22±0.10
0.25	2.47±0.19
0.5	1.4±0.15
1.0	1.85±0.04
1.5	2.15±0.06
2.0	2.15±0.06
2.5	2.35±0.12
3.0	2.3±0.04

Callus produced per leaves explant. Mean±SE (*n*=10).

**Table 1.4:** Measurement of the OD reading of control, positive control (vitamin C and BHT) and other samples taken at 515nm using spectrometer.

Samples	OD reading (515 nm)
Control (ethanol 95%)	0.058
Vitamin C	0.005
BHT	0.008
Leaves extracts (10 mg/ml)	0.017
Callus extracts (10 mg/ml)	0.031
Leaves extracts (20 mg/ml)	0.027
Callus extracts (20 mg/ml)	0.036
