# Supplementary material for: Callus cell proliferation from broccoli leaf slice using IBA and BAP in vitro culture: Its biochemical and antioxidant properties
Source: Data Brief. 2015 Dec 12;6:214–20. doi: 10.1016/j.dib.2015.11.061 (PMC4707183; doi:10.1016/j.dib.2015.11.061)
Supplement: Supplementary file 1 — Supplementary material [file mmc1.doc]

**Conflict of interest**

Dear Editor

We do not have any conflict of interest in publishing the research work in Data in Brief.

Best Regards

Dr. ABM sharif Hossain

(on behalf of all authors)
